# Is floral structure a reliable indicator of breeding system in the Brassicaceae?

**DOI:** 10.1371/journal.pone.0174176

**Published:** 2017-03-21

**Authors:** Phillip A. Salisbury, Yvonne J. Fripp, Allison M. Gurung, Warren M. Williams

**Affiliations:** 1 Faculty of Veterinary and Agricultural Sciences, University of Melbourne, Parkville, Victoria, Australia; 2 AgriBio, Centre for AgriBioscience, Department of Economic Development, Jobs, Transport and Resources, Bundoora, Victoria, Australia; 3 Department of Genetics, La Trobe University, Bundoora, Victoria, Australia; 4 AgResearch, Grasslands Research Centre, Palmerston North, New Zealand; Nanjing Agricultural University, CHINA

## Abstract

This study investigated the usefulness of floral characters as a potential indicator of breeding system in the Brassicaceae. Initially, pod set, seed set and pollen tube growth experiments were carried out to confirm the breeding systems of 53 lines representing 25 different cultivated and weedy species from the Brassicaceae. The results of the pod set tests clearly differentiated between self-compatible and self-incompatible species. Floral characters were then evaluated on one or more lines of each of the 25 species. Fourteen floral characters were evaluated including, flower diameter, Cruden’s outcrossing index, timing and direction of dehiscence and pollen-ovule ratio. Significant differences between species were evident in all of the floral characteristics evaluated. Flower diameter was generally larger in self-incompatible species than self-compatible species and pollen/ovule ratio was generally higher in self-incompatible species than self-compatible species. However, none of the floral characteristics was able to clearly differentiate the self-compatible and self-incompatible species and allow prediction of the breeding system with absolute confidence. The floral characteristic which was most effective at differentiating the two groups was anther direction at dehiscence.

## Introduction

The ability to easily predict breeding systems through evaluation of floral characteristics is beneficial to the plant breeder to enable effective crossing to be carried out and to maximise heterozygosity in outcrossing species and thus ensure effective maintenance of genetic stocks. The *Brassicaceae* family includes many important crop species including edible oil crops, vegetable crops, fodder crops and industrial oil crops [1]. In addition, the family includes over 100 weedy species which are potential sources of traits for improvement of the cultivated species [2].

Studies of the *Brassicaceae* family have found a mixture of self-compatible and self-incompatible species [3–8]. Bateman [3] noted that flower size and nectary arrangement were major factors in determining the breeding system of the *Brassicaceae*, and suggested that self-incompatibility was strongly correlated with flower size. Fryxell [4] concluded that while floral structure may be indicative of reproductive behaviour, it alone could not be relied on to determine breeding systems.

In a study of a wide range of plant families, Cruden [9] concluded that pollen/ovule ratios were a better indicator of the breeding system of the plant than either floral size or morphology. In species of the *Brassicaceae*, Preston [10] found that both the mean number of pollen grains per flower and the pollen/ovule ratio were significantly greater in outcrossing species than in self-pollinating species, with the pollen/ovule ratio the best indicator of the breeding system. Pollen/ovule ratio was also found by Takahata et al. [11] to be closely related with the breeding system of *Brassica* species and allied genera. The evolutionary transition in flowering plants from outcrossing to selfing has occurred multiple times and is generally associated with a distinctive set of floral characteristics, known as the selfing syndrome (as reviewed by Sicard and Lenhard [12]). A number of floral changes have been reported to accompany the loss of self-incompatibility, including a change from extrorse to introrse anthers in paired stamens, a decrease in the number of pollen grains per flower and a decrease in the pollen/ovule ratio associated with decreased anther length, a decrease in pistil length due to decreasing style length, and smaller petals [12–14]. These changes give populations capable of selfing an advantage over outcrossers when pollinators are scarce. Cruden [9] developed an outcrossing index, taking into account flower size and temporal and spatial separation of anthers and stigma, which related to breeding systems.

The aims of this study were to determine the breeding systems of Brassicaceae species found in Australia and to evaluate a range of floral characteristics in the species to see if any could be used to accurately predict the breeding system.

## Materials and methods

### Plant material

Fifty-three lines, representing 25 different species were used in the experiments (Table 1). The lines comprised 19 weedyBrassicaceae species collected from wild species populations found in cropping regions of Australia [15]. In addition, six commonly cultivated reference species were included. The number of lines representing each wild species was variable, depending on the availability of germplasm and the characteristic being measured. All lines were multiplied in a glasshouse at Horsham, Victoria [15].

**Table 1:** Pod and seed set in glasshouse selfing experiment in wild Brassicacae and cultivated reference species.

Species	No. lines	Pod set (%)		No. seed/pod set		% ovules resulting in seed	Breeding classification^a^
		Mean	Range	Mean	Range		
**Wild Species**							
*Brassica fruticulosa*	3	3	0–10	1	-	0.1%	SI
*Brassica oxyrrhina*	1	93		15		93.8%	SC
*Brassica tournefortii*	3	98	95–100	19	18–20	84.1%	SC
*Camelina sativa*	1	100		18		84.1%	SC
*Capsella bursapastoris*	3	100	-	24	21–27	79.5%	SC
*Carrichtera annua*	3	97	94–100	4	4–5	57.1%	SC
*Conringia orientalis*	2	97	93–100	37	36–38	76.1%	SC
*Diplotaxis muralis*	2	100	-	40	34–46	99.5%	SC
*Diplotaxis tenuifolia*	3	6	0–9	3	1–5	0.1%	SI
*Diplotaxis tenuisiliqua*	1	7		2		0.1%	SI
*Hirschfeldia incana*	3	1	0–4	2	-	22.2%	SI
*Myagrum perfoliatum*	2	97	-	1	-	50.0%	SC
*Raphanus raphanistrum*	3	0	-	-	-	-	SI
*Rapistrum rugosum*	3	0	-	-	-	-	SI
*Sinapis arvensis*	3	6	0–17	2	-	14.9%	SI
*Sisymbrium erysimoides*	2	100	-	31	30–31	58.1%	SC
*Sisymbrium irio*	3	100	-	57	51–68	97.6%	SC
*Sisymbrium officinale*	3	96	93–100	15	-	87.2%	SC
*Sisymbrium orientale*	3	100	-	118	90–147	81.6%	SC
**Cultivated reference lines**							
*Brassica rapa* cv. Bunyip	1	3		3		11.9%	SI
*Brassica carinata* CPI 100564	1	90		10		54.3%	SC
*Brassica juncea* cv. Stoke	1	43		5		23.1%	SC
*Brassica napus* cv. Tatyoon	1	100		19		70.9%	SC
*Brassica nigra* CPI 104440	1	0		-		-	SI
*Sinapis alba* cv. Kirby	1	0		-		-	SI

^a^SI—self-incompatible, SC—self-compatible

### Glasshouse selfing experiment

A bagging experiment was used to determine breeding system (self-compatibile or self-incompatibile). After germination in gibberellic acid solution, 5–10 seedlings were transplanted into 20 cm diameter pots in the glasshouse, with seedling numbers thinned to two per pot once plants were established. Four plants of each line were evaluated. Two or more inflorescences per plant, with 5–10 (or more in small flowered lines) unopened flowers per inflorescence, were bagged with small "breathable" glassine bags. Open flowers were removed from the inflorescence prior to bagging along with a small number of flower buds below the test flowers for identification purposes. Each inflorescence was tagged and the exact number of flowers bagged per inflorescence was recorded. Bags were shaken every few days to facilitate pollen movement. Where no pod development was evident, bags were left on until the styles wilted and dropped off. If pod development was evident, bags were removed when pods started to push against the bags. Regular observations were made on the number of pods developed in each inflorescence and the degree of pod development. The number of seed set per pod and the degree of development of the seed was recorded at maturity.

The pod set percentage (the proportion of pods that contained viable seed) was calculated as:

[(No. pods containing viable seed)/(No. flowers bagged)]×100

### Pollen tube growth study

Twenty-five lines, representing 19 wild crucifer species and six cultivated reference species, previously identified as either self-compatible or self-incompatible, were evaluated for pollen tube growth after self- and cross-pollination. For two plants of each species, two flowers were self-pollinated and two flowers were cross-pollinated. For both treatments, flowers were emasculated prior to anther dehiscence, making the treatments identical except for pollen type. Timing of natural dehiscence was identified in each species [15] and pollen was applied to the stigma to match the timing of natural dehiscence. For the self-pollination treatment, pollen from three flowers of the same plant was applied to the stigma. For cross-pollination, pollen from three different plants of the same line was applied to the stigma.

Twenty-four hours after pollination, the pistils were removed from the plant and treated in the following way:

1. Fixed for one hour in 3:1 ethanol:acetic acid solution.
2. Rinsed in 70% ethanol for a minimum of one hour (could be stored indefinitely at this stage).
3. Softened for 30 minutes to 5 hours (softening time required was shorter for small flowers) in 5N NaOH, then rinsed in water.
4. Stained in decolourized aniline blue overnight.

The pistils were then squashed under a coverslip and viewed under a fluorescence microscope.

### Flower diameter and outcrossing index

Flower diameters were measured on 95 lines, consisting of 1–10 lines of each wild species and the cultivated reference species. Diameters were measured at the widest point on 5–10 recently dehisced flowers of four plants from a birdcage experiment. Flowers produced very early or very late in the flowering period were excluded.

The outcrossing index of Cruden [9] was calculated by summing the mean scores for each line for the following three characteristics:

1. Flower diameter: <1 mm = 0, 1–2 mm = 1, 2–6 mm = 2, >6 mm = 3
2. Temporal (time) separation of anther dehiscence and stigma receptivity: No separation = 0, separation = 1
3. Spatial relationship of stigma and anthers: Stigma and anthers on same level and contact possible = 0, stigma and anthers spatially separated and contact unlikely = 1.

### Timing and direction of anther dehiscence

**Fig 1:**
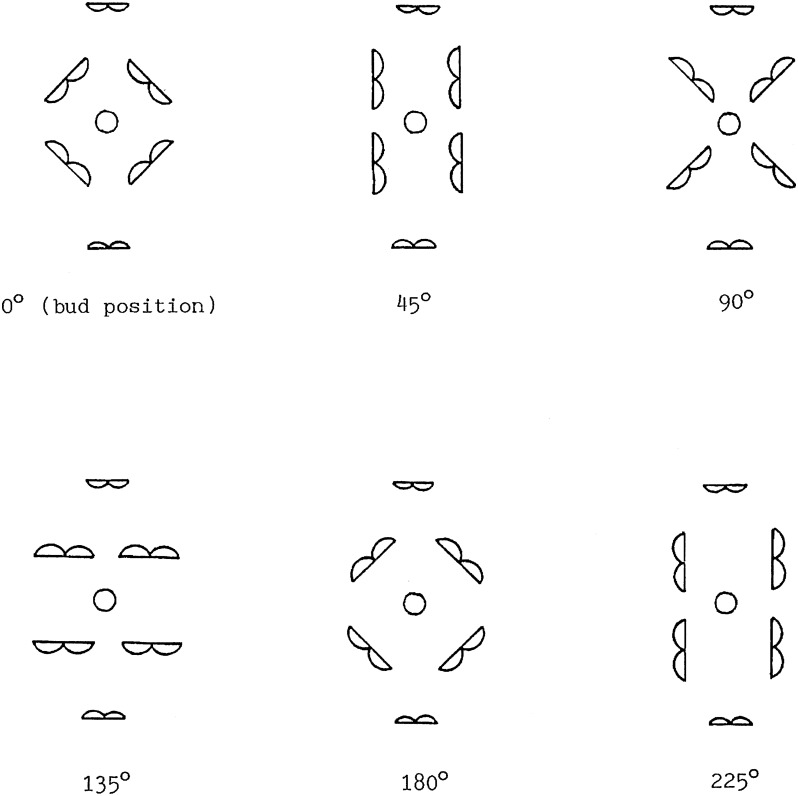
Direction of dehiscence of the paired anthers relative to their initial position in the flower bud. Central circle represents the stigma.

The timing of anther dehiscence relative to flower opening and the direction of anther dehiscence relative to the stigma was investigated in 1–3 lines of each of the wild species and the cultivated reference species. Observations were made on twenty to fifty flowers on each of five plants of each line. Results for direction of dehiscence are expressed in terms of the degree of rotation of the filaments of the paired anthers relative to their starting position in the flower bud (Fig 1).

### Pollen and ovule number and pollen/ovule ratio

The number of pollen grains and ovules per flower and the pollen/ovule ratio were determined for 25 lines, consisting of one line of each wild species and the cultivated reference species. One flower from each of five plants was evaluated for each line. Immediately prior to dehiscence, the anthers were dissected and pollen grains were teased from the anthers into a drop of 1:3 glycerol:lactic acid solution on a slide. The pollen grains were mixed evenly through the solution. A coverslip was then gently placed over the solution, enabling it to spread evenly under the entire area of the coverslip. The microscope magnification was adjusted to ensure a significant number of pollen grains (greater than 50) per quadrat. Pollen grain counts were made in at least five quadrats. The area of the quadrat was measured using an ocular micrometer and the number of pollen grains under the entire coverslip calculated. Depending on the size of the anthers, one to three anthers were counted from each flower. If the anther sacs of the tall and short anthers were of different sizes, counts were made of each type. The pollen grain number for the whole flower was then calculated.

Ovule numbers were also determined on the same flowers as used for pollen grain counts. Pistils were prepared in the manner described for the pollen tube growth study, except staining in aniline blue was not required. Ovules were counted under a binocular microscope.

For each flower, the pollen/ovule ratio was determined by dividing the number of pollen grains per flower by the number of ovules.

### Style, ovary and pistil length

Style, ovary and pistil lengths were determined in the same 25 lines used for the pollen and ovule counts. Two to five flowers from each of five plants were measured per line, using an ocular micrometer in a binocular microscope.

The style to ovary ratio was calculated for each individual flower by dividing style length by ovary length.

### Stigma width, stigma surface area and pollen grain volume

Stigma width and depth (not presented) were measured using an ocular micrometer on the same flowers used for pistil length measurements. Stigma surface area was calculated by approximating the shape to that of half an ovoid. The surface area of a sphere 4*πr*^2^ becomes 4*πr*_1_*r*_2_ for an ovoid. The calculation for stigma surface area thus became 4*πr*_1_*r*_2_/2, where r_1_ = stigma width/2 and r_2_ = stigma depth.

The length and width of the ovoid pollen grains were measured on at least five pollen grains from a single flower from each of five plants of each species. These were used to calculate pollen grain volume. The volume of a sphere $\frac{4}{3}\pi r^{3}$ becomes $\frac{4}{3}\pi r_{1}^{2}r_{2}$ for an ovoid. The volume of the pollen grains were therefore calculated as

$$\frac{4}{3}\;\pi {\left(\frac{pollen\;grain\;length}{2}\right)}^{2}\times \left(\frac{pollen\;grain\;width}{2}\right)$$

### Statistical analysis

Using the Genstat statistical package, analyses of variance were carried out for the number of pollen grains per flower, the number of ovules per flower, the pollen/ovule ratio, flower diameter, style length, ovary length, style-ovary ratio, pistil length, stigma width, stigma surface area and pollen grain volume. Principal component analysis of the data was also carried out.

Where residual graphs indicated that the variance increased with the mean, the data were log transformed, re-analysed and the least significant range (LSR) value was calculated [16]. Treatment means were significantly different if their ratio, using the largest value as the numerator, exceeded the LSR. For the flower diameter data (with multiple entries for species), where the data are presented in a summary form, the LSR is thus applicable to individual line comparisons, rather than species means.

## Results

### Glasshouse selfing experiment

The results for pod set and seed set with selfing (bagging) clearly distinguished the lines into two distinct groups, either self-compatible or self-incompatible (Table 1). All the self-compatible lines had at least 90% pod set, with the exception of *Brassica juncea*, which had 43% pod set. In comparison, the self-incompatible species had a maximum of 17% pod set, with most less than 10% and several with zero (Table 1). In the self-incompatible cultivatedspecies *Brassica rapa*, occasional small pods developed after selfing which contained no seed.

There were no major differences between different populations of the same species with respect to breeding system. The biggest difference occurred in *Sinapis arvensis*, where one population had 17% pod set, compared with zero for the other two.

When a self-incompatible plant did develop viable pods, very few seeds were produced per pod (Table 1).

### Pollen tube growth study

The results for pollen tube growth after self-pollination and cross-pollination confirmed the results of the bagging experiment (Table 2). After cross-pollination using pollen from three different plants, it was observed that in all lines, a large number of pollen grains had successfully germinated and produced pollen tubes which had moved down the style to the ovules. A similar picture was revealed after self-pollination of the lines shown to be self-compatible from the bagging study. The only exception occurred in *Brassica juncea* cv. Stoke where, despite the growth of a large number of pollen tubes down the style, some retardation of pollen grain germination was evident. In contrast, selfing of self-incompatible lines generally produced no evidence of pollen tube growth in the styles. Very few pollen grains remained attached to the stigma. For those remaining, retardation of germination of the pollen grains and short, often twisted, pollen tubes were usually evident on the stigma. In rare plants from some self-incompatible species, a small number of pollen tubes (usually less than ten) could be seen in the styles and these could be traced all the way to the ovules, with apparent fertilization. In one selfed *Diplotaxis tenuifolia* plant, more than 20 pollen tubes were evident in the style.

**Table 2:** Pollen grain germination and pollen tube development after self- and cross-pollination of wild Brassicaceae and cultivated species.

Line	Self-pollination		Cross-pollination	
	Pollen grain germination^1^	Pollen tube growth^2^	Pollen grain germination	Pollen tube growth
**Self-compatible**				
*Brassica oxyrrhina*	+	+	+	+
*Brassica tournefortii*	+	+	+	+
*Camelina sativa*	+	+	+	+
*Capsella bursapastoris*	+	+	+	+
*Carrichtera annua*	+	+	+	+
*Conringia orientalis*	+	+	+	+
*Diplotaxis muralis*	+	+	+	+
*Myagrum perfoliatum*	+	+	+	+
*Sisymbrium erysimoides*	+	+	+	+
*Sisymbrium irio*	+	+	+	+
*Sisymbrium officinale*	+	+	+	+
*Sisymbrium orientale*	+	+	+	+
*Brassica carinata* CPI 100564	+	+	+	+
*Brassica juncea* cv. Stoke	+/R	+	+	+
*Brassica napus* cv. Tatyoon	+	+	+	+
**Self-incompatible**				
*Brassica fruticulosa*	R/(+)	S	+	+
*Diplotaxis tenuifolia*	R/(+)	S	+	+
*Diplotaxis tenuisiliqua*	R	-	+	+
*Hirschfeldia incana*	R	-	+	+
*Raphanus raphanistrum*	R	-	+	+
*Rapistrum rugosum*	R	-	+	+
*Sinapis arvensis*	R	-	+	+
*Brassica rapa* cv. Bunyip	R	-	+	+
*Brassica nigra* CPI 104440	R	-	+	+
*Sinapis alba* cv. Kirby	R	-	+	+

^1^R Retarded pollen grain germination, (+) small number of germinating pollen grains, + large number of germinating pollen grains

^2^- No pollen tube growth, S small number of pollen tubes growing and fertilising ovules, + large number of pollen tubes growing and fertilising ovules

### Flower diameter and outcrossing index

**Fig 2:**
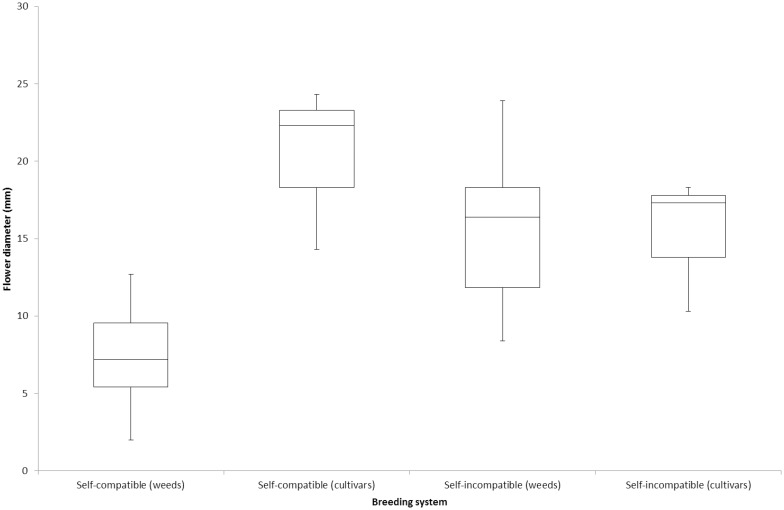
Comparison of flower diameter between self-compatible and self-incompatible species, with the median and ranges indicated.

Flower diameters in the different lines ranged from 1.7 mm to 27.3 mm (Table 3). The flower diameters of the self-incompatible species were generally considerably larger than for the self-compatible species but significant overlap between the two groups was evident (Table 3, Fig 2). The relatively large diameter of the self-compatible *Diplotaxis muralis* and the small diameter of the self-incompatible species *Hirschfeldia incana* contributed to the overlap. The cultivated self-compatible *Brassica* species also contributed to the overlap, with larger flower diameter than the self-compatible weedy species. Mean flower diameter of the cultivated self-compatible *Brassica* species ranged from 14.3 mm to 24.3 mm (Fig 2), which was comparable to the self-incompatible species.

**Table 3:** Flower diameters and outcrossing index scores in self-compatible and self-incompatible species.

Species	Flower diameter (mm)			Outcrossing index
	No. lines	Mean	Range	
**Self-compatible**				
*Brassica oxyrrhina*	1	9.7		3
*Brassica tournefortii*	8	8.7	7.7–10.3	3
*Camelina sativa*	1	5.7		2
*Capsella bursapastoris*	5	2.0	1.7–2.3	1
*Carrichtera annua*	3	9.1	7.7–10.3	3
*Conringia orientalis*	2	9.5	9.0–10.0	3
*Diplotaxis muralis*	2	12.7	12.0–13.3	3
*Myagrum perfoliatum*	2	5.2	5.0–5.3	2
*Sisymbrium erysimoides*	2	3.5	3.0–4.0	2
*Sisymbrium irio*	5	5.5	5.0–6.0	2
*Sisymbrium officinale*	7	5.7	5.0–6.3	2
*Sisymbrium orientale*	7	10.8	9.3–13.7	3
*Brassica carinata* CPI 100564	1	24.3		3
*Brassica juncea* cv. Stoke	1	14.3		3
*Brassica napus* cv. Tatyoon	1	22.3		3
**Self-incompatible**				
*Brassica fruticulosa*	5	16.4	14.7–18.3	3
*Diplotaxis tenuifolia*	7	19.2	15.3–22.7	3
*Diplotaxis tenuisiliqua*	1	12.3		3
*Hirschfeldia incana*	9	8.4	7.7–9.7	3
*Raphanus raphanistrum*	10	23.9	20.0–27.3	3
*Rapistrum rugosum*	7	11.4	10.3–13.0	3
*Sinapis arvensis*	5	17.4	15.3–19.3	3
*Brassica rapa* cv. Bunyip	1	18.3		3
*Brassica nigra* CPI 104440	1	10.3		3
*Sinapis alba* cv. Kirby	1	17.3		3
LSR (P = 0.05)	1.164			

The outcrossing index of Cruden [9] was ineffective in differentiating the two breeding types (Table 3). No temporal or spatial separation of stigma and anthers was evident in any of the species investigated, so any differences in score were exclusively due to differences in flower diameter. Scores of 1 or 2 were confined to the self-compatible group, but a score of 3 was common in both groups.

### Timing and direction of anther dehiscence

In all of the self-incompatible species anthers dehisced after the flower had opened (Table 4). All of the species where dehiscence occurred before the flower had opened significantly were self-compatible, but in some self-compatible species (*Diplotaxis muralis* and the cultivated species) dehiscence occurred after flower opening (Table 4).

**Table 4:** Timing and direction of dehiscence in self-compatible and self-incompatible species.

Species	Timing of dehiscence^1^	Direction of dehiscence
**Self-compatible**		
*Brassica oxyrrhina*	B	90°
*Brassica tournefortii*	B	45–90°
*Camelina sativa*	B	45°
*Capsella bursapastoris*	B	45°
*Carrichtera annua*	B	45°
*Conringia orientalis*	B	45°
*Diplotaxis muralis*	A	90°
*Myagrum perfoliatum*	B	90°
*Sisymbrium erysimoides*	B	45°
*Sisymbrium irio*	B	45°
*Sisymbrium officinale*	B	45°
*Sisymbrium orientale*	B	45°
*Brassica carinata* CPI 100564	A	45°
*Brassica juncea* cv. Stoke	A	90–135°
*Brassica napus* cv. Tatyoon	A	90°
**Self-incompatible**		
*Brassica fruticulosa*	A	135°
*Diplotaxis tenuifolia*	A	90–135°
*Diplotaxis tenuisiliqua*	A	135°
*Hirschfeldia incana*	A	90–135°
*Raphanus raphanistrum*	A	45°
*Rapistrum rugosum*	A	90–135°
*Sinapis arvensis*	A	135°
*Brassica rapa* cv. Bunyip	A	90–225°
*Brassica nigra* CPI 104440	A	90–135°
*Sinapis alba* cv. Kirby	A	90–135°

^1^B—before flower significantly open, A—after flower open

All species evaluated had six anthers. In all but one of the species the anther arrangement was the same, with two tall pairs of anthers and two short single anthers. The exception was *Camelina sativa*, which had one tall pair of anthers, one intermediate pair and two short single anthers. In the flower bud of all species, all anthers were introrse, or facing inwards toward the stigma. The two short single anthers in the flower of all species evaluated did not rotate and remained introrse after dehiscence. However, the degree of rotation of the filaments of the paired anthers and the consequent direction of dehiscence (Table 4) tended to be related to the breeding system. All of the self-compatible species except *Brassica juncea* were still considered introrse, with the anthers either facing inwards toward the stigma at dehiscence (45° rotation) or having rotated only 90° (Fig 1; Table 4), so that pollen transfer to the stigma was still easily possible. In contrast, in most self-incompatible species, further rotation of the anthers (90°-225°) had occurred, causing extrorse dehiscence. The only exception was *Raphanus raphanistrum*, whose paired anthers did not rotate, but remained introrse during and after dehiscence.

### Pollen and ovule number and pollen/ovule ratio

Significant differences in the number of pollen grains per flower, the number of ovules per flower and the pollen/ovule ratios were evident between species (Table 5). The estimated number of pollen grains per flower varied from 6,200 in *Capsella bursapastoris* to over 140,000 in *Raphanus raphanistrum* (Table 5). While the number of pollen grains per flower was generally lower in self-compatible compared with self-incompatible species, there were a number of exceptions (Table 5). Both *Conringia orientalis* and *Sisymbrium orientale* had a high number of pollen grains per flower, as did the three self-compatible *Brassica* cultivated species.

**Table 5:** Number of pollen grains per flower, number of ovules per flower and pollen/ovule ratios in self-compatible and self-incompatible species.

Line	No. pollen grains/flower	No. ovules/flower	Pollen/ovule ratio
**Self-compatible**			
*Brassica oxyrrhina*	43,300	16.0	2,704
*Brassica tournefortii*	18,620	22.6	825
*Camelina sativa*	10,020	21.4	471
*Capsella bursapastoris*	6,200	30.2	206
*Carrichtera annua*	13,500	7.0	1,923
*Conringia orientalis*	70,820	48.6	1,465
*Diplotaxis muralis*	21,240	40.2	526
*Myagrum perfoliatum*	15,160	2.0	7,580
*Sisymbrium erysimoides*	7,680	53.4	144
*Sisymbrium irio*	12,280	58.4	210
*Sisymbrium officinale*	12,440	17.2	723
*Sisymbrium orientale*	79,520	144.6	553
*Brassica carinata* CPI 100564	76,520	18.4	4,157
*Brassica juncea* cv. Stoke	90,940	21.6	4,201
*Brassica napus* cv. Tatyoon	106,420	26.8	3,985
**Self-incompatible**			
*Brassica fruticulosa*	80,280	20.2	3,998
*Diplotaxis tenuifolia*	99,460	58.6	1,692
*Diplotaxis tenuisiliqua*	61,600	22.5	2,739
*Hirschfeldia incana*	46,840	9.0	5,204
*Raphanus raphanistrum*	143,440	8.0	17,897
*Rapistrum rugosum*	39,000	2.0	19,500
*Sinapis arvensis*	110,080	13.4	8,269
*Brassica rapa* cv. Bunyip	63,500	25.2	2,622
*Brassica nigra* CPI 104440	86,225	13.0	6,621
*Sinapis alba* cv. Kirby	92,240	5.8	16,004
LSR (P = 0.05)	1.251	1.134	1.217

Mean ovule numbers per flower ranged from 2.0 (*Myagrum perfoliatum*, *Rapistrum rugosum*) to 144.6 (*Sisymbrium orientale*). No trend was evident between the breeding groups for the number of ovules per flower, with both self-compatible and self-incompatible species ranging from very low to high numbers (Table 5).

**Fig 3:**
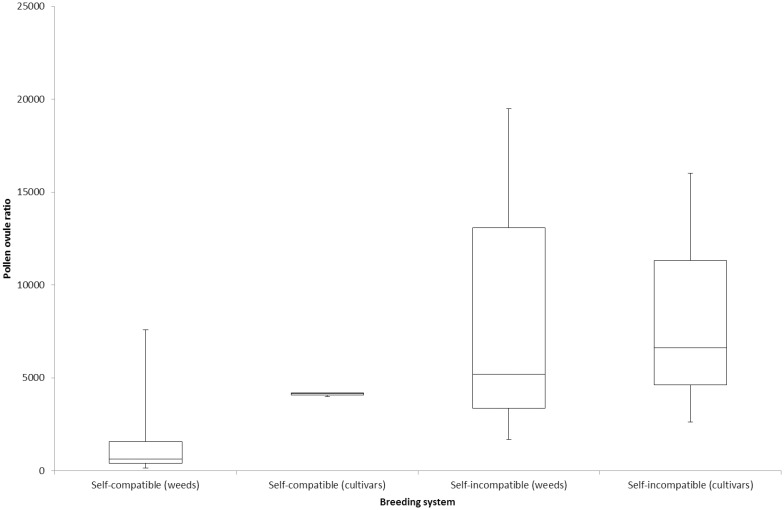
Comparison of pollen ovule ratio between self-compatible and self-incompatible species, with the median and ranges indicated.

Pollen/ovule ratios varied from 144 in *Sisymbrium erysimoides* to 19,500 in *Rapistrum rugosum*. The pollen/ovule ratios were generally lower in the self-compatible species, although *Myagrum perfoliatum* and the cultivated species were exceptions (Table 5, Fig 3). *Diplotaxis tenuifolia* had a pollen/ovule ratio much lower than the rest of the self-incompatible species.

For both pollen grains per flower and pollen/ovule ratios, there was considerable overlap between the two breeding groups (Fig 3).

### Style, ovary and pistil length

Significant differences in style, ovary and pistil lengths were found between species (Table 6). Style lengths ranged from 0.05 mm in *Sisymbrium officinale* to 3.85 mm in *Brassica juncea* and *Raphanus raphanistrum*. While a number of self-compatible species had very short style lengths, there was again some overlap between the two breeding groups. The longer styles of self-compatible species *Brassica oxyrrhina* and the cultivated Brassica species and the relatively short style of *Diplotaxis tenuisiliqua* were the cause of overlap. The pattern for pistil length was similar to that of style length.

**Table 6:** Style, ovary and pistil length in self-compatible and self-incompatible species.

Line	Style length (mm)	Ovary length (mm)	Style length /ovary length	Pistil length (mm)
**Self-compatible**				
*Brassica oxyrrhina*	2.90	3.02	0.96	6.06
*Brassica tournefortii*	1.20	3.01	0.40	4.48
*Camelina sativa*	0.61	2.06	0.30	2.88
*Capsella bursapastoris*	0.21	0.91	0.23	1.26
*Carrichtera annua*	1.98	1.15	1.86	3.35
*Conringia orientalis*	0.23	4.30	0.05	4.85
*Diplotaxis muralis*	1.52	4.22	0.36	6.44
*Myagrum perfoliatum*	0.88	1.16	0.78	2.21
*Sisymbrium erysimoides*	0.14	2.37	0.06	2.67
*Sisymbrium irio*	0.11	1.79	0.07	2.11
*Sisymbrium officinale*	0.05	2.28	0.02	2.49
*Sisymbrium orientale*	0.23	4.76	0.05	5.30
*Brassica carinata* CPI 100564	2.59	5.64	0.46	8.84
*Brassica juncea* cv. Stoke	3.85	3.77	1.04	8.10
*Brassica napus* cv. Tatyoon	3.05	5.64	0.54	9.15
**Self-incompatible**				
*Brassica fruticulosa*	1.96	4.30	0.46	6.66
*Diplotaxis tenuifolia*	1.71	6.51	0.27	9.11
*Diplotaxis tenuisiliqua*	0.97	3.44	0.29	4.58
*Hirschfeldia incana*	3.60	1.78	2.07	5.76
*Raphanus raphanistrum*	3.85	6.97	0.59	10.86
*Rapistrum rugosum*	2.37	1.92	1.25	4.62
*Sinapis arvensis*	3.28	2.18	1.56	5.84
*Brassica rapa* cv. Bunyip	2.14	3.58	0.60	6.09
*Brassica nigra* CPI 104440	1.91	3.32	0.58	5.68
*Sinapis alba* cv. Kirby	2.62	1.67	1.63	4.76
LSR (P = 0.05)	1.272	1.183	1.298	1.146

No real trend was evident between the breeding groups for ovary length, which ranged from 0.91 mm (*Capsella bursapastoris*) to 6.97 mm (*Raphanus raphanistrum*). Major overlap was observed in the ranges of the self-compatible and the self-incompatible species.

The style length-ovary length ratio was very low in a number of self-compatible species, especially the *Sisymbrium* species and *Conringia orientalis*, but again did not clearly differentiate between the two groups.

### Stigma width, stigma surface area and pollen grain volume

The values for stigma width, stigma surface area and pollen grain volume in *Diplotaxis muralis* (self-compatible) and *Diplotaxis tenuifolia* (self-incompatible), were considerably larger than all other species (Table 7). While the lowest values for stigma width, stigma surface area and pollen grain volume were in self-compatible species, considerable overlap between the two breeding groups was observed (Table 7).

**Table 7:** Stigma width, stigma surface area and pollen grain volume in self-compatible and self-incompatible species.

Line	Stigma width (mm)	Stigma surface area (mm^2^)	Pollen grain volume (mm^3^x10^-4^)
**Self-compatible**			
*Brassica oxyrrhina*	0.509	0.473	0.225
*Brassica tournefortii*	0.545	0.494	0.245
*Camelina sativa*	0.328	0.202	0.195
*Capsella bursapastoris*	0.293	0.128	0.084
*Carrichtera annua*	0.218	0.110	0.195
*Conringia orientalis*	0.739	0.846	0.138
*Diplotaxis muralis*	1.402	3.096	0.632
*Myagrum perfoliatum*	0.363	0.192	0.141
*Sisymbrium erysimoides*	0.353	0.182	0.119
*Sisymbrium irio*	0.403	0.255	0.105
*Sisymbrium officinale*	0.313	0.165	0.133
*Sisymbrium orientale*	0.684	0.678	0.141
*Brassica carinata* CPI 100564	0.938	1.827	0.283
*Brassica juncea* cv. Stoke	0.833	1.266	0.286
*Brassica napus* cv. Tatyoon	0.894	1.298	0.262
**Self-incompatible**			
*Brassica fruticulosa*	0.730	0.932	0.316
*Diplotaxis tenuifolia*	1.226	2.415	0.379
*Diplotaxis tenuisiliqua*	0.635	0.593	0.181
*Hirschfeldia incana*	0.603	0.731	0.222
*Raphanus raphanistrum*	0.499	0.642	0.176
*Rapistrum rugosum*	0.541	0.567	0.249
*Sinapis arvensi*	0.713	0.811	0.371
*Brassica rapa* cv. Bunyip	0.648	0.758	0.165
*Brassica nigra* CPI 104440	0.715	1.041	0.221
*Sinapis alba* cv. Kirby	0.873	1.260	0.344
LSR (P = 0.05)	1.142	1.270	1.079

### Overall comparison

A comparison of the results in the self-compatible and self-incompatible species for all floral characteristics measured was made using cut-off values based on the minimum value observed for each trait in the self-incompatible species (Table 8). This confirmed that none of the characteristics completely differentiated the two breeding groups. However, a number of the characteristics partially differentiated the two groups. The characteristics which most effectively differentiated the groups were anther direction at dehiscence and timing of dehiscence. The number of ovules per flower was totally ineffective at differentiating the two groups, with ovary length also extremely ineffective.

**Table 8:** Comparison of floral characters in self-compatible and self-incompatible species.

Floral character	Values	No. species	
		Self-incompatible	Self-compatible
Pollen grains/flower	<39,000	0	9
	≥39,000	10	6
Ovules/flower	<2	0	0
	≥2	10	15
Pollen/ovule ratio	<1,692	0	9
	≥1,692	10	6
Flower diameter (cm)	<8.4	0	6
	≥8.4	10	9
Outcrossing index	1–2	0	6
	3	10	9
Timing of dehiscence	Before flower opens	0	11
	After flower opens	10	4
Anther direction at dehiscence	≤45°	1	14
	>45°	9	1
Style length (mm)	<1.45	0	8
	≥1.45	10	7
Ovary length (mm)	<1.71	0	3
	≥1.71	10	12
Style/ovary length	<0.36	0	5
	≥0.36	10	10
Pistil length (mm)	<4.38	0	7
	≥4.38	10	8
Stigma width (mm)	<0.499	0	7
	≥0.499	10	8
Stigma surface area (mm^2^)	<0.567	0	9
	≥0.567	10	6
Pollen grain volume (mm^3^x10^-4^)	<0.165	0	7
	≥0.165	10	8

**Fig 4:**
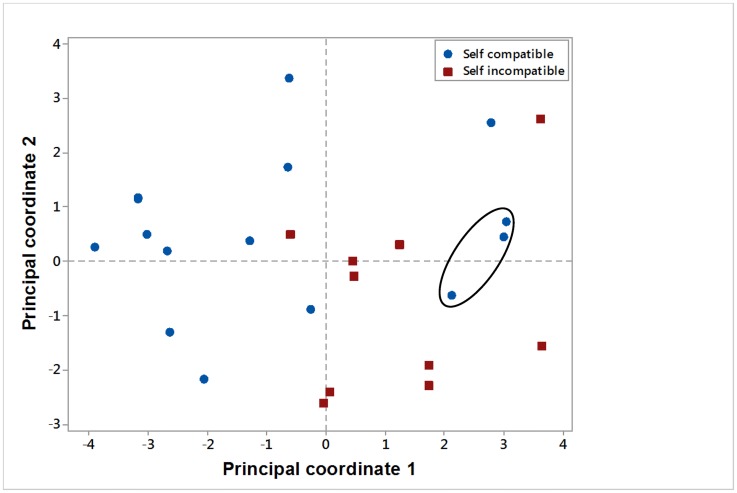
Principal coordinate analysis plot of the 25 Brassicaceae species based on 11 floral characters. The self-compatible cultivated reference lines *B*. *napus*, *B*. *juncea* and *B*. *carinata* are indicated within the oval.

**Fig 5:**
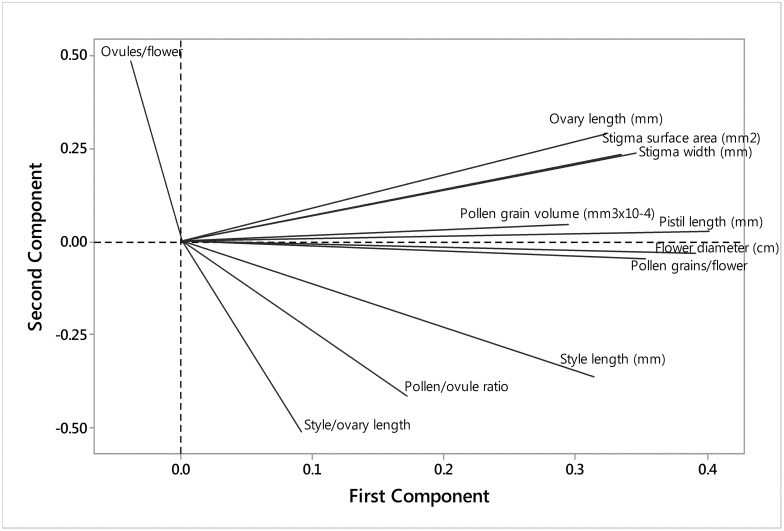
Principal coordinate analysis loading plot of the 11 floral characters for the Brassicaceae species.

Principal component analysis was carried out on all 11 continuous floral characters. Seventy four percent of the total variation was extracted by the first two components. Fig 4 is a scatter diagram of the scores for the first two components. There was a distinct separation between many of the self compatible and self incompatible species. However, there were a few species that did not group according to breeding system. In particular, the self compatible cultivated species *B*. *napus*, *B*. *juncea* and *B*. *carinata* grouped with most of the self incompatible species. Relationships between the 11 floral characters are displayed in Fig 5. Many of the characters grouped together, with ovules/flower the most distinct from the other characters.

## Discussion

### Selfing experiment and pollen tube growth

All the species tested were clearly categorized as either self-compatible or self-incompatible. Attempts to self pollinate self-incompatible species almost always produced inhibition of germination, with short, often twisted pollen tubes barely penetrating the stigmatic surface. These results showed that the stigmatic surface was the site of inhibition of pollen grain germination in all of the self-incompatible wild species (*Brassica fruticulosa*, *Diplotaxis tenuifolia*, *Diplotaxis tenuisiliqua*, *Hirschfeldia incana*, *Raphanus raphanistrum*, *Rapistrum rugosum* and *Sinapis arvensis*) and self-incompatible cultivated species (*Brassica rapa*, *Brassica nigra* and *Sinapis alba*) examined. This agrees with previous observations in the wild species including *Capsella grandiflora* [17], *Iberis amara* [18] and *Leavenworthia* [13] and in cultivated species of the *Brassicaceae* [19–21].

The results for the pollen tube growth studies clearly confirmed the results of the bagging experiment. Lines which produced little or no seed on selfing invariably showed retardation of pollen grain germination with little or no pollen tube development. Lloyd [13] similarly showed a close relationship between pollen tube growth tests and silique growth tests in a study of self-compatible and self-incompatible races of two *Leavenworthia* species.

In the self-incompatible species *Brassica fruticulosa* and *Diplotaxis tenuifolia*, a small number of pollen tubes were observed growing down the style and apparently fertilizing the ovules. This correlates with the occasional occurrence of a small number of seeds in selfed pods, as was found in these two species and other self-incompatible species. The appearance of a few pollen tubes which penetrated the stigma, grew through the style rapidly and were capable of effecting fertilization was reported by Johnson [21] in self-incompatible *Brassica oleracea*. Within population differences between plants in the strength of their self-incompatibility reaction have been noted by Lloyd [13], Johnson [21], Richards and Thurling [22] and Hinata and Nishio [23]. Examination of more plants within a line and more lines of each species would be valuable to gain a greater insight into the degree of intra species variation for the self incompatibility trait.

In addition to genetic differences between plants, several environmental factors have also been shown to affect the expression of self-incompatibility in the *Brassicaceae*. These include high temperatures [21, 22, 24], flower age [25] and stage of flowering [21]. Hinata and Nishio [23] concluded that although self-incompatibility is principally controlled by S alleles, any population is a composite of heterogeneous plants with different manifestations of incompatibility which may be balanced among several genetic and environmental factors. Given such factors, the growth of a small number of pollen tubes and the appearance of occasional seed in a few pods of the self-incompatible species, as observed in this study, is not unexpected.

### Flower diameter and outcrossing index

Bateman [3] concluded that self-incompatibility was strongly correlated with flower size in the *Brassicaceae*, with self-incompatible plants generally having bigger and brighter flowers. Likewise, larger and more attractive flowers were reported in self-incompatible races relative to self-compatible races of *Leavenworthia* [13]. Hinata and Nishio [23] found that the self-compatible species *Brassica tournefortii* and *Brassica oxyrrhina* had smaller flowers than other self-incompatible *Brassica* species. While the flower diameter of self-incompatible species in the current study was generally larger than for self-compatible species, large flowers were not restricted to the self-incompatible group. The degree of overlap between the self-compatible and self-incompatible groups in this study was such that flower diameter was an imprecise indicator of breeding system, thus supporting similar conclusions by Fryxell [4] and Cruden [9]. The four largest flowered self-compatible species (*Diplotaxis muralis* and the cultivated lines) were also the only self-compatible species which dehisced after flower opening. This was probably not coincidental, with insect pollination likely to be important in species which dehisce after flower opening and large flowers required to better attract insects.

The outcrossing index of Cruden [9] was of little use in predicting the breeding system of the *Brassicaceae* species evaluated. The crossing experiments in this study and that of Bateman [3] did not reveal any protandry (male organ maturing first) or protogyny (female organ maturing first) in crucifer species, indicating no temporal separation with respect to dehiscence of pollen and the receptivity of the stigma. Likewise, no spatial separation was evident in this study, with the height of the paired anthers virtually level with the stigma in all species. All the differences in outcrossing index were thus due entirely to differences in flower diameter. This index seems more suited to comparisons across a range of plant families, rather than within a family such as the *Brassicaceae*.

### Timing and direction of dehiscence

Cruden [9] reported that the flowers of facultative autogamous species tended to self-pollinate prior to or during flower opening, and that the exposure of receptive stigmas to potential pollinators occurred after self-pollination had occurred. A number of self-compatible species in this study likewise dehisced before the flowers were significantly open. However, other self-compatible species, namely *Diplotaxis muralis* and the cultivated *Brassica* species, dehisced after flower opening, as did all the self-incompatible species. Thus while dehiscence prior to flower opening is associated with self-compatibility, dehiscence after flower opening is not exclusively associated with self-incompatibility. Timing of dehiscence relative to flower opening cannot therefore accurately predict the breeding system of all species.

In all of the species in the current study, the single anthers did not rotate, but remained introrse. This has previously been reported in *Leavenworthia* [13] and cultivated *Brassica* species [26, 27].

In contrast to the single anthers, the filaments of the paired anthers tended to slowly turn so that the anthers were rotated to a degree characteristic of each species. Rajan [26] reported that dehiscence in paired anthers of self-compatible cultivated *Brassica* species was introrse, while rotation in paired anthers of self-incompatible cultivated *Brassica* species resulted in extrorse dehiscence. The trend across the range of species evaluated in this study was not as clear cut. With the possible exception of *Brassica juncea*, all the self-compatible species were more or less introrse, having rotated no more than 90° at the time of dehiscence. Within these self-compatible species, however, two different types were present. In some species, the anthers of the paired stamens always remained introrse, in others they eventually rotated to become extrorse. Hinata and Nishio [23] commented on these extremes of the self-compatible spectrum within *Brassica*. Lloyd [13] even found some self-compatible races of *Leavenworthia* which were extrorse at the time of dehiscence.

With one exception, the self-incompatible species in this study had rotated more than 90° and were extrorse at the time of dehiscence. The exception was *Raphanus raphanistrum*, whose anthers remained introrse and did not rotate beyond 45°. This would seem inefficient for a self-incompatible species, where pollen from a different plant is required for successful pollination, and does not appear to have been reported previously.

Given such exceptions, and the fact that degree of rotation can be affected by weather and that sometimes differences are observed between different anthers in the same flower [13], it is clear that direction of dehiscence alone cannot be used to precisely predict breeding systems in the *Brassicaceae*.

### Pollen and ovule number and pollen/ovule ratio

A comparison of the pollen grain and ovule numbers and the pollen/ovule ratios in the 13 species which were common to this study and those of Hinata and Konno [6], Cruden [9] and Preston [10] showed general agreement in the results, although some differences were evident. The number of pollen grains in *Brassica rapa* cv. Bunyip was considerably less than found in this species by Preston [10], but was in the range presented by Hinata and Konno [6] for *Brassica rapa*. Fewer ovules per flower were reported by Preston [10] for *Brassica nigra*, *Sinapis arvensis* and *Sisymbrium orientale* than observed in this study, with fewer pollen grains also for the latter two. Results from the current study would suggest that in smaller flowers of any given species, both pollen grain and ovule numbers can be reduced, thereby having little effect on pollen/ovule ratio. Hinata and Konno [6] found fewer pollen grains in late flowers of *Brassica rapa*.

While the number of pollen grains per flower in self-compatible species was often lower than for self-incompatible species in this study and those of Lloyd [13], Hinata and Konno [6] and Preston [10], there was considerable overlap between the two breeding groups in all cases. It seems that while a low number of pollen grains per flower is indicative of self-compatibility, higher pollen grains numbers can occur in both breeding groups. The self-compatible species identified with high pollen grain numbers tend to be those which dehisce after flower opening.

Although there were significant differences between species in number of ovules per flower, as there were no differences between the two breeding groups for this character in this study or in Lloyd [13] and Preston [10], it could not be used to differentiate between them.

While the species with the lowest pollen/ovule ratios in this study were self-compatible, major overlap in the range of pollen/ovule ratios for the self-compatible and self-incompatible groups was found. One self-compatible species, *Myagrum perfoliatum*, had a pollen/ovule ratio of 7,580, higher than many of the self-incompatible species. Even though Lloyd [13], Cruden [9], Preston [10] and Takahata et al. [11] all claimed that pollen/ovule ratios were good indicators of the breeding system, considerable overlap between breeding groups was likewise evident in their studies. The results of the current study have confirmed that pollen/ovule ratios cannot be used to predict the breeding system with absolute confidence.

### Style, ovary and pistil length

Values and rankings for style, ovary and pistil length for species in this study were generally similar to those reported in the Subtribe *Brassicinae* by Takahata and Hinata [7] and in *Capsella bursapastoris* by Cruden and Lyon [28]. Major exceptions were a much longer style in *Brassica juncea* and a much shorter style in *Sinapis alba* in this study relative to that of Takahata and Hinata [7], although ovary lengths were similar in both studies. Observations from this study indicate that any change in style length due to conditions at flowering or stage of flowering is accompanied by a corresponding change in ovary length, resulting in a similar style length/ovary length ratio. The observed differences between the studies would therefore seem to be due to genetic differences between the lines, rather than environmental effects.

A decrease in pistil length due to a decrease in style length accompanied the loss of self-incompatibility in races of *Leavenworthia* [13]. While all the species with very short styles in this study were self-compatible, a number of self-compatible species also had longer styles, causing style length and pistil length to be imprecise indicators of breeding system. Ovary length in this study was of no use in predicting breeding system, consistent with the results of Takahata and Hinata [7].

While the style length/ovary length ratio can be used as a distinguishing characteristic for different species [7], it could not be used to differentiate between self-compatible and self-incompatible species. Although the species with the lowest style length/ovary ratio were self-compatible, major overlap between the two groups was evident.

### Stigma width, stigma surface area and pollen grain volume

It could be postulated that in self-incompatible species, the stigma width or the stigma surface area might need to be larger than in self-compatible species, in order to give a larger target for the pollen grains required from other plants for successful fertilisation. No such relationship was evident in the results, however, with significant overlap between the two groups for both stigma width and stigma surface area. These characteristics were useful, however, for distinguishing some species. *Diplotaxis muralis* and *Diplotaxis tenuifolia* were the only species with a stigma width greater than 1 mm. Takahata and Hinata [7] had likewise found that *Diplotaxis* species were the only ones where the stigma width exceeded 1 mm.

The pollen grain volume of *Capsella bursapastoris* in the current study was somewhat larger than that reported by Cruden and Lyon [26], resulting from a radius approximately 1.5 times bigger. No other values were found for comparison. The results of Cruden and Miller-Ward [29] indicated a trade-off between pollen grain number and size across a range of families and a strong negative correlation between pollen/ovule ratio and pollen surface area, indicating the possibility of a relationship between pollen grain size and breeding system. However, no such relationship was evident, within the *Brassicaceae*, with major overlap between the two breeding groups in pollen grain volume.

### Overall comparison

None of the floral characteristics evaluated could be used individually to predict the breeding systems of the different lines with complete accuracy. Principal component analysis with 11 of the floral characters demonstrated that a clear distinction of the breeding system for every species cannot be achieved even when multiple floral characters are taken into account. For many characteristics, the self-compatible cultivated species, *Brassica carinata*, *Brassica juncea* and *Brassica napus*, were a major cause of the overlap between the self-compatible and the self-incompatible groups. These species generally had bigger flowers and floral parts than the other self-compatible species. This is consistent with Takahata and Hinata [7] and Takahata et al. [11], who pointed out that the cultivated plants had been derived from self-incompatible species having larger organs and were again selected in the direction of having larger organs through breeding by man. Evolutionary transition in flowering plants from self-incompatible to self-compatible has occurred multiple times and is accompanied by changes to the floral characteristics, including smaller flowers and less pollen grains per ovule [12]. The presence of floral characters typical of a self-incompatible species in a self-compatible species may indicate that the transition to self compatibility has been very recent, as observed in *Linaria cavanillesii* by Voillemot and Pannell [30].
